# Directly observed therapy at opioid substitution facilities using sofosbuvir/velpatasvir results in excellent SVR12 rates in PWIDs at high risk for non-adherence to DAA therapy

**DOI:** 10.1371/journal.pone.0252274

**Published:** 2021-06-04

**Authors:** Caroline Schmidbauer, Michael Schwarz, Angelika Schütz, Raphael Schubert, Cornelia Schwanke, Enisa Gutic, Roxana Pirker, Tobias Lang, Thomas Reiberger, Hans Haltmayer, Michael Gschwantler

**Affiliations:** 1 Department of Internal Medicine IV, Klinik Ottakring, Vienna, Austria; 2 Division of Gastroenterology and Hepatology, Department of Internal Medicine III, Medical University of Vienna, Vienna, Austria; 3 Vienna HIV & Liver Study Group, Vienna, Austria; 4 Ambulatorium Suchthilfe Wien, Suchthilfe Wien gGmbH, Vienna, Austria; 5 Sigmund Freud University, Vienna, Austria; University of Sassari, ITALY

## Abstract

**Background & aims:**

We evaluated the effectiveness of sofosbuvir/velpatasvir (SOF/VEL) in difficult-to-treat PWIDs with presumed high risk for non-adherence to antiviral therapy using an innovative concept involving their opioid agonist therapy (OAT) facility.

**Methods:**

N = 221 patients (m/f: 168/53; median age: 44.7 years (IQR 16.9); HCV-genotype 3: 45.2%; cirrhosis: 33.9%) treated with SOF/VEL were included. PWIDs at high risk for non-adherence to DAA therapy (n = 122) received HCV treatment alongside OAT under the supervision of medical staff ("directly observed therapy", DOT). These patients were compared to patients with presumed excellent drug compliance, who were treated in a "standard setting" (SS) of SOF/VEL prescription at a tertiary care center (n = 99).

**Results:**

DOT-patients (n = 122/221; 55.2%) were younger than SS-patients (median age: 41.3 vs. 53.0 years), all had psychiatric comorbidities and most had a poor socioeconomic status. 83/122 (68.0%) reported ongoing intravenous drug use. Within the DOT-group, SVR12 was achieved in 99.1% (95% CI: 95.0–100; n = 109/110) with one patient experiencing treatment failure, while n = 12/122 (9.8%) patients were excluded due to loss of follow-up (FU). 5 patients showed HCV reinfection after achieving SVR12. SS-patients achieved SVR in 96.6% (95% CI: 90.3–99.3%; n = 84/87) after exclusion of 10/99 (10.1%) patients who were lost to FU and 2 patients who died prior to SVR12 due to reasons not related to DAA therapy.

**Conclusions:**

SOF/VEL given as DOT along with OAT in PWIDs at high risk of non-adherence to antiviral therapy including those with ongoing intravenous drug use resulted in excellent SVR rates similar to patients with presumed “excellent compliance” under standard drug intake.

## Introduction

Hepatitis C virus (HCV) infection represents one of the major causes for liver disease and HCV-associated cirrhosis used to be among the main indications for liver transplantation worldwide. Therefore, the World Health Organization (WHO) has declared global HCV elimination an international public health goal [1–3]. In order to achieve this, both increasing HCV diagnosis rates from <5% (2015) to 90% (2030) and treatment rates from <1% (2015) to 80% (2030) of all eligible patients are necessary [3–6].

While some countries such as Egypt and Pakistan are currently on track to reach these goals, most European countries are still working on improving their HCV elimination strategies [7–10]. Targeted screening programs aiming at specific key population groups (e.g. prison inmates and people who inject drugs—PWIDs) have been conducted in many countries, yet linkage to care often remains the most difficult factor in these HCV elimination scenarios [11–22].

While the historic HCV treatment with pegylated interferon showed insufficient cure rates and a high rate of adverse events, modern pangenotypic direct acting antiviral agents (DAA) result in high sustained virologic response (SVR) rates and a favorable drug safety profile in almost all patients [18, 20, 22–28]. This includes patients who used to be considered "difficult-to-treat populations" such as pretreated subjects, HIV/HCV coinfected patients and PWIDs [18, 20, 29–35].

One of the pangenotypic DAA combinations currently used in Europe is sofosbuvir/velpatasvir (SOF/VEL). SOF/VEL is a fixed-dose combination tablet of sofosbuvir, an inhibitor of the NS5B RNA polymerase blocking viral replication, and velpatasvir, which inhibits the NS5A protein required for the assembly and release of viral particles. SOF/VEL proved to be safe and showed SVR12 rates close to 100% in previous studies [36–38]. Since the removal of reimbursement restrictions concerning HCV treatment in Europe, access to DAA including SOF/VEL has been simplified for all patients [39]. However, real world data on SOF/VEL are still scarce, especially regarding special risk groups like PWIDs [22, 37, 38, 40].

Numerous phase III studies investigating the effectiveness of pangenotypic DAA have been performed recently [22]. PWIDs–and especially those with ongoing injection drug use (IDU)—were mostly excluded from these studies, however, this subpopulation represents one of the most important risk groups for HCV infection and transmission, especially because they are often unaware of their HCV-infection [14, 15, 17, 41–43]. Psychiatric comorbidities and unstable socioeconomic circumstances are common in PWIDs—especially those with ongoing IDU. Hence, linkage to HCV care and ensuring therapy adherence pose pivotal challenges in this patient collective and high-prevalence group. A tailored treatment approach is necessary in order to include PWIDs in international HCV elimination plans [15, 42–45]. Thus, we assessed the effectiveness of an innovative concept of directly observed therapy (DOT) in PWIDs with presumed poor adherence and drug compliance, i.e. providing SOF/VEL therapy along regularly dispensed opioid agonist therapy (OAT) at their respective substitution facilities.

## Patients & methods

### Study design and treatment setting

This study was performed as a retrospective analysis of routinely acquired clinical data in a defined cohort of HCV-infected patients. We included all patients aged 18 years or older who started treatment with sofosbuvir/velpatasvir (SOF/VEL) for chronic hepatitis C between September 17th 2016 and October 14th 2019. Both DAA-naïve and pretreated patients who had completed the 12 week follow-up period after end of therapy were included. Due to the retrospective design of the study, no sample size calculation was performed. Treatment was initiated via one of the Viennese HCV treatment centers, i.e. a tertiary care hospital collaborating with a low-threshold drug-addiction facility in Vienna.

The decision for SOF/VEL as a treatment regimen was made in the clinical routine setting and based on liver function (i.e. prevalence of decompensated cirrhosis), comedication (i.e. contraindications due to drug-drug-interactions between patients’ long-term medication and alternative HCV-treatment regimens), pretreatment (i.e. history of HCV-treatment with alternative HCV-treatment regimens) and patients’ nutritional habits (i.e. problems ensuring the ingestion of the HCV-medication with or right after a meal). Conversely, treatment duration, specific drug-drug-interactions, potential side effects and—where applying—the combination with ribavirin and associated clinical consequences were evaluated and discussed with each patient.

Currently, there are about 8.000 PWIDs living in Vienna who are included in a national OAT program [46]. These patients receive their daily OAT on a regular basis at a pharmacy or at a low-threshold facility to ensure each OAT-patient ingests their own dose of medication. According to estimations, about 30% of this population are infected with HCV [44].

In order to overcome the barrier of linkage to HCV care for PWIDs, we established a cooperation between the tertiary care center Klinik Ottakring and the Ambulatorium Suchthilfe Wien. Ambulatorium Suchthilfe Wien is a low threshold drug addiction treatment facility offering integrative care for PWIDs. This includes medical services such as hepatological and psychiatric counseling, OAT distribution and support by social workers. To facilitate linkage to care, an outpatient hepatitis clinic directed at PWIDs was established at the Ambulatorium Suchthilfe Wien. At this institution PWIDs were seen by an experienced hepatologist and pretreatment evaluation, like laboratory tests and transient elastography (Fibroscan®), were conducted. Thus, we combined HCV screening and treatment for PWIDs in a multidisciplinary setting located in a low-threshold institution specifically aimed at PWIDs.

**Fig 1:**
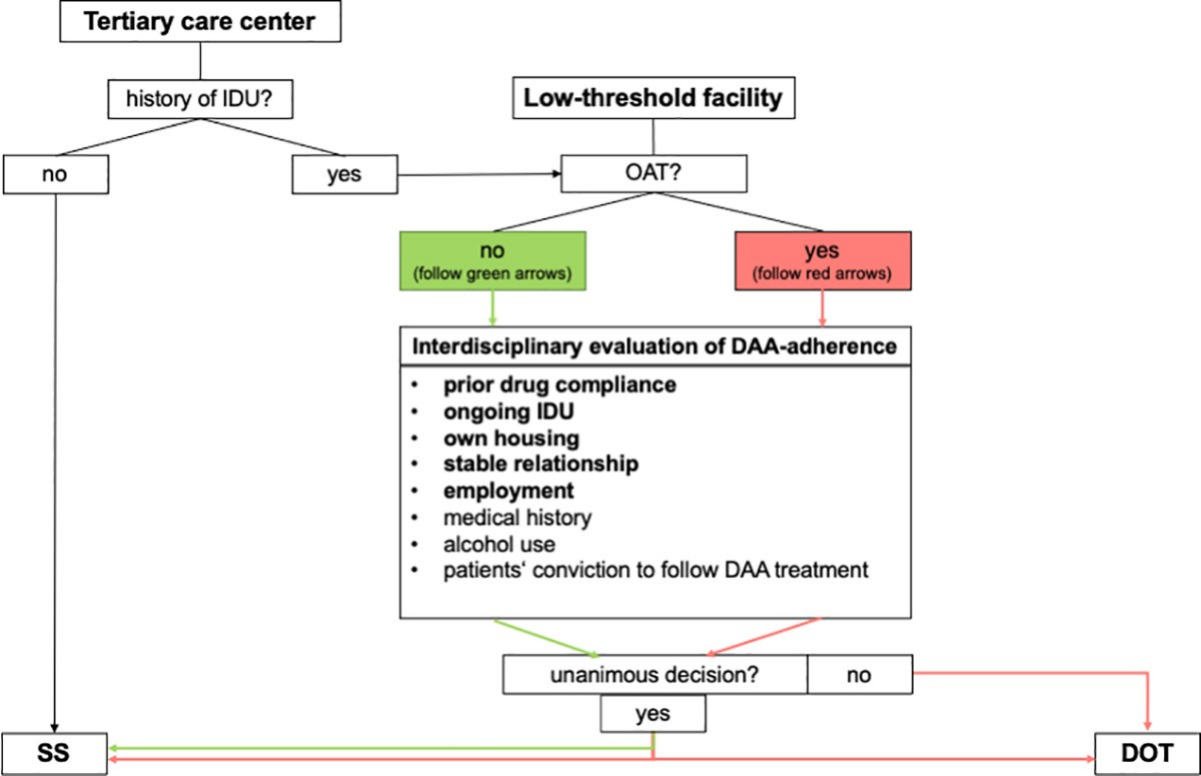
Flowchart describing the process of assigning patients to the directly observed therapy-group (DOT) or "standard setting" (SS). Abbreviations: IDU, intravenous drug use; OAT, opioid agonist therapy; DAA, direct acting antivirals; SS, standard setting; DOT, directly observed therapy. Patients without a history of intravenous drug use (IDU) presenting at the tertiary care center were assigned to the SS-group. Also, patients with a history of IDU and presumed excellent compliance concerning adherence to DAA-therapy presenting at the tertiary care center or at the low-threshold facility were treated according to the SS. Patients on OAT with presumed high risk of non-adherence to DAA-therapy according to a unanimous decision between the members of the interdisciplinary evaluation team were assigned to the DOT-group. In case of different opinions among the evaluation team members concerning patients’ presumed compliance, the DOT setting was selected.

Besides a hepatologist, patients were also seen by addiction medicine specialists and the institution’s head nurse. This interdisciplinary team evaluated the probability of reliable, self-administered HCV medication intake for each patient (Fig 1). Factors that were taken into account during this evaluation process included the individual patient’s medical history and prior drug compliance concerning other indications if available, and the current socioeconomic situation of the patient along with the individual patient’s conviction to be able to strictly follow the instructions for DAA treatment, and the presence of ongoing IDU. Patients’ socioeconomic status was assessed by analyzing the following factors: 1) alcohol use (if present, this was taken as an indicator towards non-adherence to DAA), 2) employment status (unemployment was taken as an indicator towards non-adherence to DAA), 3) own housing (homelessness or lack of own housing were taken as indicators towards non-adherence to DAA), 4) stable relationship (lack thereof was taken as an indicator towards non-adherence to DAA), 5) criminal record (a history of imprisonment was taken as an indicator towards non-adherence to DAA). During the compliance evaluation process, the highest priority was attributed to previous and/or current drug compliance concerning other indications (prior bad compliance was taken as an indicator towards non-adherence to DAA), the presence of ongoing IDU (ongoing IDU was taken as an indicator towards non-adherence to DAA), and the presence of stable housing and social support as assessed by the presence of a stable relationship and employment. The remaining factors served as supporting information. Patients in whom the prioritized factors pointed towards non-adherence to DAA and those in whom one priority factor and one or more supporting factor pointed towards non-adherence to DAA were considered at risk for non-adherence to DAA. The decision on the individual treatment setting for each patient was based on the decision of the multidisciplinary evaluation team, predicating on an individual voting system among the team members. Since this evaluation system was significantly based on clinical assessment, patients were considered at risk for non-adherence to DAA if DAA adherence was doubted by one or more members of the interdisciplinary team despite the assessment of the mentioned factors. Patients in whom therapy adherence was unanimously estimated to be low, were assigned to our concept of “directly observed therapy” (DOT): To optimize adherence, antiviral therapy was handed to patients together with their OAT to be ingested under direct observation by a pharmacist, physician or nurse at a pharmacy or at the Ambulatorium Suchthilfe Wien on a once-daily (i.e. daily from Monday to Saturday, only the doses for Sunday were handed to the patients in advance for self-administration at home) or, in some selected cases, once-weekly, twice-weekly or thrice-weekly basis (“directly observed therapy group”, DOT). In cases of questionable adherence and/or heterogeneous opinions within the multidisciplinary team, the corresponding patients received SOF/VEL according to the concept of DOT. In patients treated according to the concept of DOT, the frequency of DAA-dispensation was determined according to the preceding frequency of OAT-dispensation (daily vs. once, twice or three times a week): No changes were made in this schedule to keep the previously well-established routine of directly observed OAT-distribution. Thereby we hoped to increase DAA adherence and, hence, HCV cure as PWIDs might forget the ingestion of their daily DAA dose but would not forget to pick up their OAT.

Since HCV-infected PWIDs represent a very specific subgroup of patients considering their often limited compliance regarding medical treatment and procedures, we aimed to put the data from the DOT-group into context by providing similar data from a different patient collective who received treatment with SOF/VEL at our center (Klinik Ottakring): for patients without a history of IDU and PWIDs who were considered to have excellent compliance, a prescription for antiviral therapy was issued at the outpatient clinic of Klinik Ottakring once a month and the patients were only seen for routine laboratory tests at the outpatient clinic ("standard setting group", SS). This concept is the current standard of care for HCV-treatment in an outpatient setting in Austria and represents the routine approach in patients without an evident need for an individualized treatment concept at our institution. The SS group was not designed for direct comparison to the DOT group and hence patients were neither randomized nor re-assessed for their compliance. Their data are merely presented to provide an insight into drug-adherence and treatment-efficacy in a standard setting in the same geographical region and at a similar time as the DOT group and are only meant to put the results of the DOT group into perspective.

The SS group represents the patient collective treated at our outpatient clinic at a hepatological tertiary care center. Per definition, these patients independently manage their individual appointments at our institution, pick up their prescribed medication at the pharmacy and show up for scheduled check-ups. Therefore, they fulfill the prioritized criterion of current compliance and were considered to have excellent compliance concerning the planned DAA therapy as well. Socioeconomic characteristics were collected in the same manner as for the DOT group in order to provide a comprehensive characterization of the SS group.

### Pretreatment workup of patients

Pretreatment evaluation of the study population included a detailed medical history, physical examination, abdominal sonography, standard laboratory testing, serum HCV-RNA quantification, HCV genotype (GT) assessment and determination of liver fibrosis stage by transient elastography and was performed at Klinik Ottakring or at the Ambulatorium Suchthilfe Wien. The applied methods were described in detail in a previous publication of our group [15, 47]. The socioeconomic status was characterized by recording relationship status, housing status, employment status, criminal record and alcohol consumption habits.

In patients whose liver stiffness could not successfully be determined by transient elastography, fibrosis evaluation was performed by calculation of the APRI score (applied ULN for AST: 35 IU/L (female) and 50 IU/L (male); F0/F1: APRI≤0.5, F4: APRI>1.5) [15, 42, 48–50].

### Definition of ongoing intravenous drug use and measures to prevent reinfection

Ongoing IDU was defined as one or more injection within the three months preceding the initiation of HCV therapy as reported by the patient. No additional screening measures were taken to assess recent drug use. Patients with self-reported ongoing IDU received information on harm-reduction measures and prevention of HCV transmission as well as reinfection upon each visit at the low-threshold facility (e.g. needle and syringe exchange programs, hygiene measures for drug paraphernalia, etc.). Patients treated according to the concept of DOT were seen at follow-up visits every six months and received repetitive HCV-testing for reinfection after achievement of SVR.

### Antiviral therapy

Patients received one fixed-dose combination tablet of SOF/VEL (i.e. 400mg sofosbuvir and 100mg velpatasvir) daily for a treatment duration of 12 weeks, independent of cirrhosis status. At the beginning of the study addition of ribavirin to SOF/VEL was recommended in selected patients, therefore 12 of the 221 patients (all of them included in the SS-group) received a combination of SOF/VEL and ribavirin. In the event of a patient missing the ingestion of one of their daily SOF/VEL doses, the treatment period was prolonged for one day. Therefore, by the end of therapy, all patients had ingested the same originally calculated number of SOF/VEL tablets.

### Study endpoints

The primary endpoint of this study was SVR, defined by an HCV-RNA level <15 IU/ml 12 weeks after end of treatment (SVR12). SVR12 rates were calculated for all patients who received at least one dose of DAA and for the patients who received at least one dose of DAA excluding patients who failed to achieve SVR12 for reasons other than virological failure; e.g. patients lost to FU and patients who died due to reasons not related to therapy before SVR12 was confirmed. Patients with poor adherence to therapy who missed DAA-ingestion on one or more days but were not lost to FU were included in the analyses of SVR rates after LTFU (lost to follow-up) exclusion.

Adherence to DAA in PWIDs treated in the DOT setting as well as the frequency of early termination of treatment and the occurrence rate of serious adverse events were assessed as secondary endpoints.

### Statistics

Median (IQR, interquartile range) and numbers (with percentages) of a specific characteristic were used to describe continuous variables and categorical variables, respectively. The calculation of *p-values* was performed using Student’s t-test, Wilcoxon-Mann-Whitney-test or chi-squared test, depending on the type of variable and the presence of normal distribution. Proportions of each fibrosis-stages and HCV genotypes were compared between groups by chi-squared test resp. Fisher´s exact test according to case numbers. Two-sided 95% exact confidence intervals (CI) were calculated using the Clopper-Pearson method.

All statistical analyses were performed using the programs Microsoft Excel for Mac 2011, Version 14.7.1; IBM SPSS Statistics, Version 25 for Mac and Prism 8 for macOS, Version 8.1.2 (227), 2019.

### Ethical considerations

The study protocol was approved by the institutional review board (Ethikkommission der Stadt Wien, EK 16-098-VK) and conducted according to the Declaration of Helsinki, Good Clinical Practice guidelines, and local regulatory requirements. Due to the strict anonymous analysis of patient data, the ethics committee waived the need for specific informed consent. However, to be absolutely sure to comply with data protection regulations, written informed consent was obtained from all patients.

## Results

### Study population

Overall, 221 HCV-infected patients were included in this study, 168 (76.0%) were male and the median age (IQR) was 44.7 (16.9) years. Five (2.3%) suffered from HIV coinfection, 75 (33.9%) had cirrhosis and 32 (14.5%) had been treated for HCV before. The most frequently detected GT were GT1 (n = 102; 46.2%) and GT3 (n = 100; 45.2%) (Table 1).

**Table 1:** Baseline characteristics.

Variable	Overall	SS	DOT	*p-value*
n (%)	221 (100)	99 (44.8)	122 (55.2)	
OAT [n (%)]	139 (62.9)	17 (17.2)	122 (100)	*<0*.*0001*
HIV [n (%)]	5 (2.3)	0 (0.0)	5 (4.1)	*0*.*066*
Sex [n (%)]				*0*.*001*
Male	168 (76.0)	65 (65.7)	103 (84.4)	
Female	53 (24.0)	34 (34.3)	19 (15.6)	
Age (years) [median (IQR)]	44.7 (16.9)	53.0 (15.7)	41.3 (11.6)	*<0*.*0001*
Fibrosis stage^†^ [n (%)]				*0*.*499*
F0/F1	61 (27.6)	29 (29.3)	32 (26.2)	*0*.*722*
F2	51 (23.1)	26 (26.3)	25 (20.5)	*0*.*394*
F3	34 (15.4)	12 (12.1)	22 (18.0)	*0*.*306*
F4	75 (33.9)	32 (32.3)	43 (35.2)	*0*.*754*
HCV genotype [n (%)]				*0*.*001*
1^‡^	102 (46.2)	48 (48.5)	54 (44.3)	*0*.*855*
• 1a	71 (32.1)	26 (26.3)	45 (36.9)	*0*.*104*
• 1b	30 (13.6)	22 (22.2)	8 (6.6)	*0*.*003*
2	4 (1.8)	4 (4.0)	0 (0.0)	*0*.*043*
3^§^	100 (45.2)	37 (37.4)	63 (51.6)	*0*.*045*
4^§^	10 (4.5)	8 (8.1)	2 (1.6)	*0*.*048*
5	1 (0.5)	1 (1.0)	0 (0.0)	*0*.*458*
not specified	5 (2.3)	2 (2.0)	3 (2.5)	*1*
Treatment experienced [n (%)]	32 (14.5)	19 (19.2)	13 (10.7)	*0*.*073*

Abbreviations: SS, standard setting; DOT, directly observed therapy; OAT, opioid agonist therapy; HIV, human immunodeficiency virus; IQR, interquartile range; HCV, hepatitis C virus.

^†^ according to transient elastography (TE; n = 195) or APRI (n = 26; applied ULN for AST: 35 IU/L (female) and 50 IU/L (male)): F0/F1: TE 0–7.1 kPa or APRI ≤ 0.5; F2: TE 7.2–9.4 kPa; F3: TE 9.5–12.4 kPa; F4: TE ≥ 12.5 or APRI > 1.5

^‡^ subtype not specified in n = 1/221 (0.5%) patient who was included in the DOT-group

^§^ including n = 1/221 (0.5%) patient with concomitant GT3/GT4 infection who was included in the SS-group

### Treatment groups

A total of 122 (55.2%) PWIDs on OAT with suspected high risk for non-adherence to HCV therapy were treated according to the concept of directly observed therapy (DOT-group) (Table 1): 110/122 patients received antiviral therapy with SOF/VEL along with their OAT at a pharmacy and 12/122 at Ambulatorium Suchthilfe Wien. Among the 122 patients who were assigned to the DOT-group, 63 (51.6%) had initially presented at the tertiary care center and 59 (48.4%) had initially presented at the low-threshold facility. 99 (44.8%) patients without a history of IDU and PWIDs with presumed excellent compliance were treated at the outpatient clinic of Klinik Ottakring, a tertiary care hospital in Vienna, Austria (standard setting group, SS).

### Comparison of clinical features

Patients who received treatment according to the concept of DOT were significantly younger and the percentage of men was higher as compared to the SS-group. The prevalence of GT3 was significantly higher and the prevalence of GT1b was significantly lower in the DOT group as compared to the SS group. 5 HIV-coinfected patients were included in the DOT group, while all patients in the SS group were HIV negative.

### Comparison of socioeconomic characteristics

Most PWIDs included in the DOT group were characterized by a poor socioeconomic status: 104 (85.2) were unemployed, 45 (36.9) reported to have no own housing and 67 (54.9) had been imprisoned before (Table 2). Only 38 (31.1) were living in a stable relationship while 83 (68.0) reported ongoing intravenous drug use (IDU). Relevant psychiatric comorbidities were prevalent in all DOT patients. Patients in the SS group reported a higher percentage of frequent alcohol consumption while the percentage of patients with own housing was higher and the percentage of patients who had been imprisoned before was lower, as compared to the DOT group (Table 2).

**Table 2:** Socioeconomic characteristics of study population at baseline.

Variable	Overall	SS	DOT	*p-value*
n (%)	221 (100)	99 (44.8)	122 (55.2)	
Alcohol abuse [n (%)]				*0*.*021*
Yes	55 (24.9)	32 (32.3)	23 (18.9)	
No	166 (75.1)	67 (67.7)	99 (81.1)	
Employment status [n (%)]				*0*.*212*
Employed	37 (16.7)	20 (20.2)	17 (13.9)	
Unemployed	182 (82.4)	78 (78.8)	104 (85.2)	
unknown	2 (0.9)	1 (1.0)	1 (0.8)	
Own housing [n (%)]				*0*.*015*
Yes	150 (67.9)	76 (76.8)	74 (60.7)	
No	67 (30.3)	22 (22.2)	45 (36.9)	
unknown	4 (1.8)	1 (1.0)	3 (2.5)	
Living in stable relationship [n (%)]				*0*.*088*
Yes	80 (36.2)	42 (42.4)	38 (31.1)	
No	138 (62.4)	56 (56.6)	82 (67.2)	
unknown	3 (1.4)	1 (1.0)	2 (1.6)	
Criminal record [n (%)]				*<0*.*0001*
Imprisoned before	94 (42.5)	27 (27.3)	67 (54.9)	
Not imprisoned before	123 (55.7)	71 (71.7)	52 (42.6)	
unknown	4 (1.8)	1 (1.0)	3 (2.5)	

### Treatment outcome and effectiveness of SOF/VEL therapy in the DOT group

**Fig 2:**
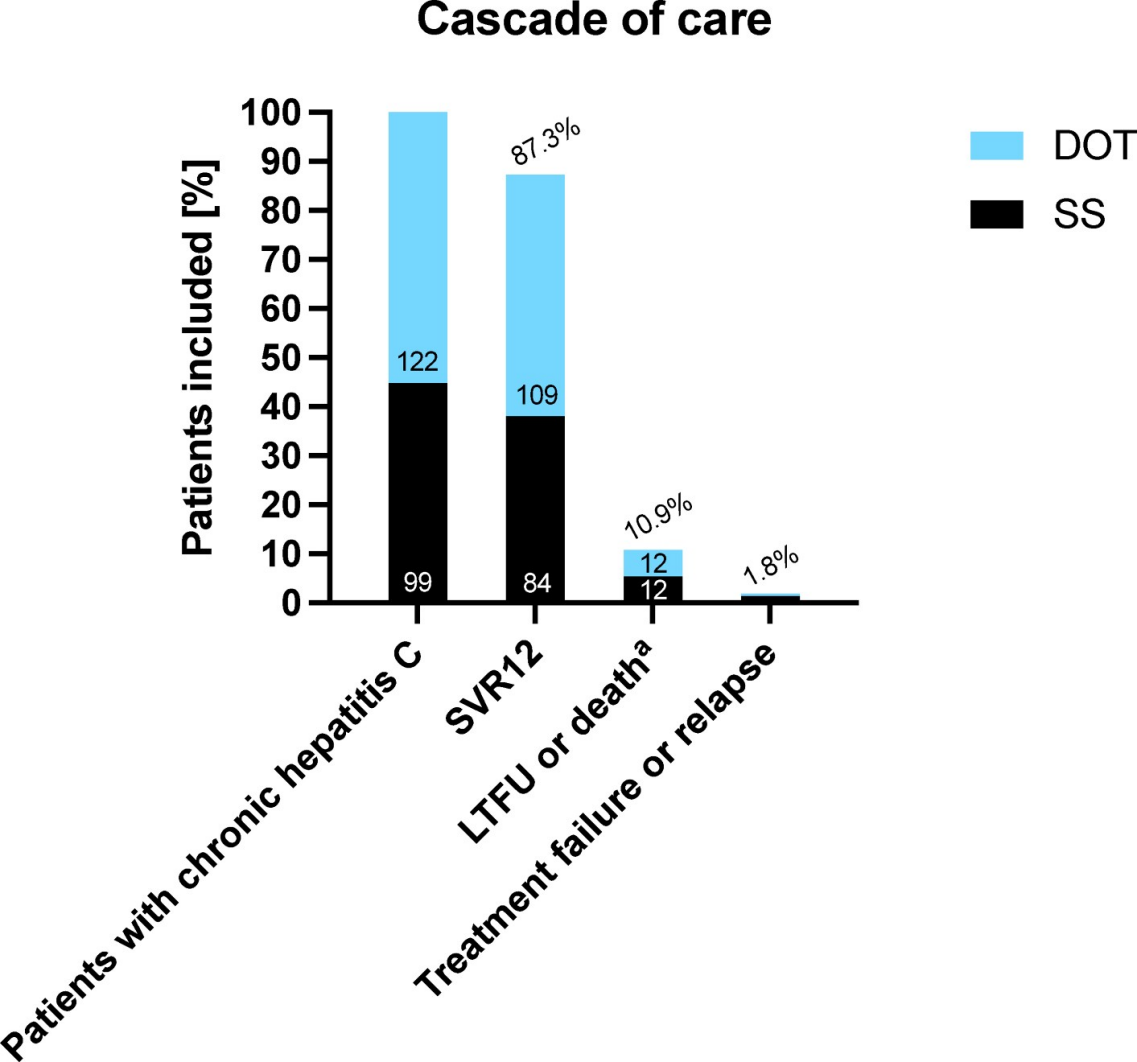
Cascade of care. Abbreviations: SS, standard setting; DOT, directly observed therapy; SVR, sustained virologic response; LTFU, lost to follow-up. ^a^ for reasons not related to therapy.

The cascade of care is summarized in Fig 2 [51]. In the DOT group, 109/122 (89.3%; 95% CI: 82.5–94.2%) patients achieved SVR12 according to the analysis of SVR rates before LTFU exclusion. Twelve (9.8%) patients were lost to FU, in one of whom end of treatment response was documented. One (0.8%) female patient with GT3a infection and fibrosis stage F4 was counted as treatment failure in our analyses: She presented with HCV viremia 25 weeks after end of treatment after SVR4 was documented. Since the patient missed the 12 week FU visit and, hence, SVR12 could not be verified, the recurrent viremia was counted as treatment failure in our analyses (genotyping could not be performed as the patient did not show up again at our center). The patient reported ongoing IDU and therefore it seems very likely that in fact SVR12 was achieved but HCV reinfection occurred due to sharing of drug application paraphernalia.

**Fig 3:**
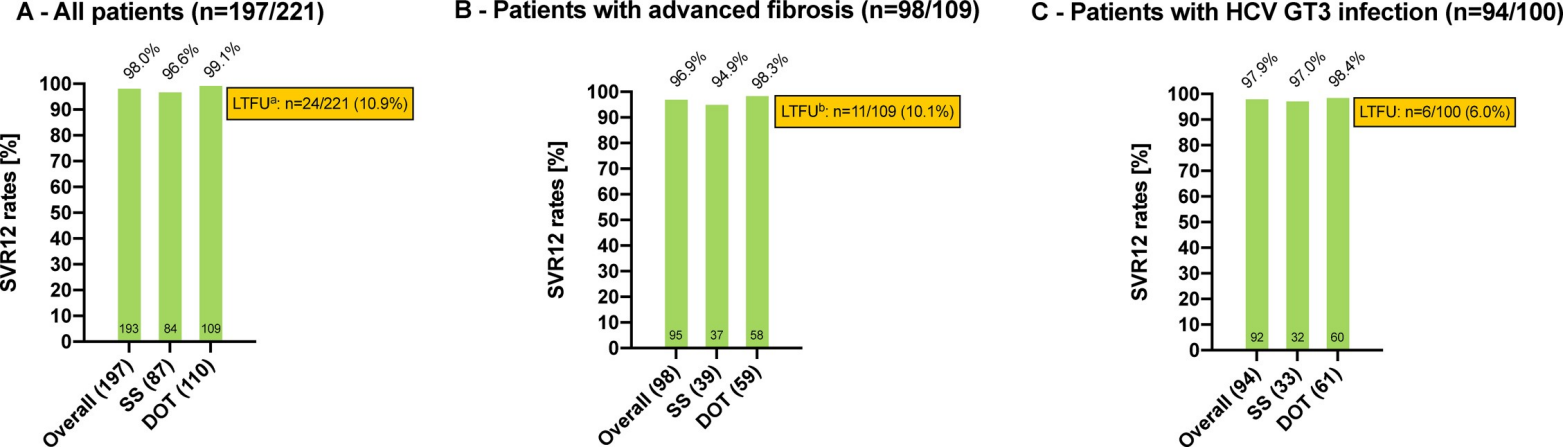
SVR12 rates after treatment with SOF/VEL after LTFU exclusion. Abbreviations: SVR, sustained virologic response; SOF/VEL, sofosbuvir/velpatasvir; LTFU, lost to follow-up; SS, standard setting; DOT, directly observed therapy; HCV, hepatitis C virus; GT, genotype. **A—All patients (n = 197/221)**. ^a^ including n = 2 patients who died for reasons not related to treatment after showing a negative HCV-RNA PCR result at week 2 of therapy and at the end of treatment, respectively. **B—Patients with advanced fibrosis (n = 98/109)**. Advanced fibrosis was defined as F3-F4: ≥9.5 kPa according to transient elastography. ^b^ including 1 patient who died for reasons not related to treatment after showing a negative HCV-RNA PCR result at week 2 of therapy. **C—Patients with HCV GT3 infection (n = 94/100)**.

After exclusion of the 12 patients who were lost to FU, SVR12 was achieved in 109/110 (99.1%; 95% CI: 95.0–100) patients according to the analysis of SVR rates after LTFU exclusion (Fig 3A–3C).

### Treatment outcome and effectiveness of SOF/VEL therapy in the SS group

In the SS group, 84/99 (84.8%; 95% CI: 76.2–91.3%) patients achieved SVR12 according to the analysis of SVR rates before LTFU exclusion, including 8 of the 12 patients who received a combination of SOF/VEL and ribavirin. Ten (10.1%) patients were lost to FU, in 4 of them end of treatment response was documented while 2 achieved SVR4. Two patients died for reasons not related to therapy before the outcome at post-treatment week 12 could be documented, one of whom had shown a negative HCV-RNA PCR at the end of treatment.

Three patients experienced a relapse within 12 weeks after end of treatment: One of these subjects was an oncological patient with decompensated cirrhosis due to HCV GT1b and chronic alcohol consumption. Treatment with SOF/VEL was started prior to the initiation of chemotherapy for gastric cancer to avoid viral flare upon immunosuppression. Surgical gastrectomy was performed during the SOF/VEL treatment period. The patient showed a negative HCV RNA PCR at week 3 and 6 of therapy and at the end of treatment. At the 12 week FU visit after end of treatment recurrent viremia was detected. The other 2 male patients who experienced virological relapse under treatment with SOF/VEL were infected with GT 1a and 3, respectively, and showed fibrosis stage F0/1 and F3, respectively. None of the 3 patients in the SS group who showed virological relapse had been treated for HCV before and none of them received additional ribavirin treatment, following the current recommendations at the time of treatment and virological relapse. Due to compliance reasons resistance-associated variants could not be assessed as the patients did not show up for further diagnostics again.

After exclusion of the patients lost to FU and the two patients who died, treatment with SOF/VEL in the SS led to SVR12 in 84/87 (96.6%; 95% CI: 90.3–99.3%) patients according to the analysis of SVR rates after LTFU exclusion (Fig 3A–3C).

### Comparison of treatment outcome

While the SS group consisted of PWIDs on OAT (n = 17/99, 17.2%) as well as patients without a history of IDU (n = 82/99, 82.8%), SVR12 rates within the SS group did not differ between these two subgroups of patients: SVR12 was achieved in 14/17 (82.4%; 95% CI: 56.6–96.2%) PWIDs and in 70/82 (85.4%; 95% CI: 75.8–92.2%) patients without a history of IDU according to the analysis of SVR rates before LTFU exclusion (*p-value 0*.*753*); 14/14 (100%; 95% CI: 76.8–100%) PWIDs and 70/73 (95.9%; 95% CI: 88.5–99.1%) patients without a history of IDU achieved SVR12 according to the analysis of SVR rates after LTFU exclusion (*p-value 0*.*440*).

Importantly, a total of 109/122 (89.3%) patients included in the DOT-group vs. 84/99 (84.8%) patients included in the SS-group achieved SVR12 according to the analysis of SVR rates before LTFU exclusion, and 109/110 (99.1%) patients included in the DOT-group vs. 84/87 (96.6%) patients included in the SS-group achieved SVR12 according to the analysis of SVR rates after LTFU exclusion. Neither the analysis of SVR rates before nor after LTFU exclusion showed a significant difference between the two groups (*p-values* for both analyses: *0*.*32*).

### Adherence to therapy and early termination of therapy

Among the 122 patients included in the DOT-group, 92 received their antiviral therapy along with OAT on a once-daily basis, 22 on a once-weekly basis, 6 on a twice-weekly and 2 on a thrice-weekly basis. Overall adherence to therapy was excellent: 113/122 (92.6%) of the patients kept all their appointments for DAA dispensation along with OAT; 4 patients missed 1 date, 3 patients missed 2 dates, and one patient each missed 3 and 4 dates, respectively. Of these 9 patients, 6 achieved SVR12. Overall, only 17 of 7104 (0.2%) scheduled dates for DAA-dispensation were missed by the 122 patients. No cases of treatment discontinuation due to adverse events or lack of compliance occurred.

### Serious adverse events

No serious adverse events related to antiviral therapy for HCV were observed in our study cohort of n = 221 patients.

### Follow-up and HCV reinfections

After a median FU (IQR) of 17 (15.0) weeks, 5 reinfections were documented in the DOT-group. The 5 reinfections occurred at week 12, 24, 28, 33 and 47 after end of therapy. In 4 of the 5 patients who showed HCV reinfection, HCV genotyping was performed: 2 patients who were initially infected with GT3a showed GT1a-reinfection and 1 patient who was initially infected with GT1a showed GT3a-reinfection. One patient who was initially infected with GT3a showed GT3a-reinfection after achieving SVR12 and SVR15. One patient who was initially infected with GT3a and for whom SVR12 was documented after treatment with SOF/VEL did not receive genotyping upon reinfection due to compliance reasons. Upon the patient’s next visit, spontaneous clearance had already occurred, therefore genotyping could not be performed. All 5 patients reported ongoing IDU, therefore reinfection due to HCV-contaminated drug injection paraphernalia was assumed in all 5 cases of HCV reinfection.

Among the 5 reinfected patients, 2 were successfully retreated for HCV with another course of SOF/VEL and glecaprevir/pibrentasvir (GLE/PIB), respectively, while 1/5 achieved spontaneous clearance. The patient who was retreated with GLE/PIB presented with another reinfection at week 56 after end of treatment with GLE/PIB and is currently on another course of treatment with SOF/VEL. In the patient who achieved spontaneous clearance after HCV-reinfection, reinfection was first detected by qualitative point-of-care HCV-RNA PCR at the low-threshold facility. The patient did not show up for further diagnostics including HCV-genotyping or quantitative HCV-RNA PCR, therefore quantitative analysis of HCV viral load could not be performed. Upon the patient’s next visit, spontaneous HCV-clearance had already occurred without HCV-active treatment. The remaining 2/5 patients with HCV-reinfections did not show up at either of our institutions again, therefore no retreatment could be initiated.

N = 44/104 (42.3%) patients assigned to the DOT group who were eligible for post-treatment week 24-evaluation by the time of data analysis did not keep their appointments for their 24 weeks FU visit. Hence, we cannot exclude that some reinfections were missed.

One of the three patients among the SS group who did not achieve SVR12 was successfully retreated with sofosbuvir/velpatasvir/voxilaprevir. The oncological patient with surgical gastrectomy who experienced treatment failure in the SS group did not receive retreatment due to the poor overall prognosis. The remaining two patients who showed treatment failure (one each in the SS and in the DOT group) could not be retreated as they did not show up at either of our institutions again.

## Discussion

The effectiveness of DAA and their excellent safety profile have been demonstrated in many studies and lately more and more data support their use in PWIDs [17, 18, 20, 22, 37, 40, 44]. However, PWIDs with ongoing IDU and a high risk of non-adherence to therapy are still excluded from many studies. Therefore, data concerning this key population remain scarce [17, 22].

In Austria, HCV treatment can only be initiated by authorized hepatological centers in the setting of tertiary care institutions. Since the removal of former national reimbursement policies, many HCV-infected patients have been treated with DAA successfully [15, 32]. However, HCV prevalence remains high among the 8.000 PWIDs in Vienna who are included in the nationwide OAT program. Therefore, measures targeting this subgroup need to be applied in order to reduce HCV-transmission [14].

Aside from effective disease-specific treatment for HCV, general actions to limit transmission of HCV and other blood-borne viruses are essential. These include needle and syringe exchange services, medical and social counseling as well as linkage to OAT [11, 12]. An important institution offering integrative care for PWIDs in Vienna is Suchthilfe Wien. The corresponding outpatient establishment, Ambulatorium Suchthilfe Wien, is a low threshold drug-treatment facility that combines HCV screening and therapy for PWIDs by providing hepatological, psychiatric and social support services as well as OAT distribution in a multidisciplinary setting [52]. Using Ambulatorium Suchthilfe Wien as an interface to connect with PWIDs who would otherwise not get linked to HCV care proved to be effective in previous studies. While PWIDs, due to their frequently prevalent psychiatric comorbidities and precarious socioeconomic situations, may not easily be linked to tertiary care medical institutions, they show an excellent adherence regarding regular visits to their pharmacy or the Ambulatorium Suchthilfe Wien to pick up their OAT. Dispensing DAA treatment for HCV along with OAT according to the concept of directly observed therapy proved to generate excellent SVR12-rates and was well accepted by PWIDs in previous studies of our group [15, 42, 43, 53].

Aside from representing a potential link to HCV-treatment for PWIDs, OAT also appears to have a relevant impact on HCV-reinfection: According to a recent meta-analysis from Australia, ongoing IDU is a major risk factor for the occurrence of HCV-reinfections after treatment-induced SVR [54]. However, linkage to OAT seemed to be a positive prognostic factor as fewer patients from this subgroup showed reinfections according to this meta-analysis [54].

Yet, a number of PWIDs with ongoing IDU are not included in an OAT program and would neither attend a hospital nor a low-threshold institution. These patients represent an especially difficult-to-treat subgroup as they show very poor compliance concerning medical treatment, while at the same time they are an important key population group with a high potential of viral transmission. Unfortunately, these patients currently cannot be treated—neither in a standard setting nor in the DOT-setting described in this study—as they are not linked to any medical institution like hepatological centers, pharmacies or low threshold facilities.

While numerous HCV-elimination projects are ongoing all over the world, real world data on SVR12 rates under pangenotypic DAA treatment are still scarce and high rates of patients LTFU are a relevant limitation in many studies, especially in projects reporting data on PWIDs. Cousien et al. for example observed a LTFU rate of 14% per year in their harm reduction program including treatment for HCV in PWIDs in France in 2017 [11], and Macías et al. reported a LTFU rate of 17% during treatment among ongoing injection drug users in their study analyzing response to DAA treatment in PWIDs with and without opioid agonist therapy in 2019 [33].

In this project, we were able to show the results of one of the first real-life analyses investigating the effectiveness of SOF/VEL: Treatment with SOF/VEL according to the DOT-concept led to an SVR12-rate of 89.9% in PWIDs at high risk of non-adherence to DAA therapy with a high prevalence of ongoing IDU. The results were comparable to those in patients with suspected good compliance and/or without a history of IDU who were treated according to the current standard of care at the outpatient clinic of a tertiary care hospital (SVR12 84.8%). Neither of the two treatment groups showed any relevant adverse events. While some bigger studies may have found even higher SVR12-rates, our findings mainly concur with current literature and support the recommendations of broad access to DAA treatment, especially for high-risk populations [12, 17–20, 22, 37, 38, 44, 45, 55, 56]. Still, bigger sample sizes will be needed to validate our results.

While the prevalence of ongoing IDU was high (68.0%) among patients in the DOT-group, the reinfection rate (4.5%) remains within the lower range as compared to current literature [11–13, 15, 54, 57]. This might in part be attributable to the fact that integrative care for PWIDs is available and accessible through low-threshold institutions in Austria. All 5 patients who presented with HCV-reinfection reported ongoing IDU.

Our study aims to support the recommendation of broad HCV-treatment including at-risk populations with special needs in order to facilitate adherence to therapy and, hence, SVR. This is one of few studies including PWIDs with ongoing IDU and showing real world data on SOF/VEL [17, 20, 22, 38]. However, there are some limitations. First, this is a monocenter study representing a selected population of PWIDs living in Vienna. Due to the differences in environment between the two groups, homogeneity in baseline characteristics, e.g. age and sex of the included patients is not provided. Furthermore, no sample size calculation was performed for this retrospective study. In order to validate the results acquired in this numerically limited cohort of patients, bigger multicenter studies and prospective study designs are needed. Also, HCV elimination in specific risk groups takes a tailored, individual approach. Depending on the individual situation and infrastructure, other cities may need to adapt our strategy and find their individual concept for providing HCV care for PWIDs taking regional frame conditions into account. A further limiting factor of this study is the individual selection of the treatment setting (DOT vs. SS): Each patient was seen by a multidisciplinary team, which, following a discussion among the team members, decided upon the ideal treatment setting for the individual patient (DOT vs. SS). Although the decision was based on a team discussion, subjective assessment of each individual patient’s presumed compliance was the key factor for the assignment of PWIDs to the treatment groups: Patients who were considered unlikely to regularly ingest their DAA if handed to them for self-administration at home were included in the DOT group. Even though the assignment of PWIDs with presumed poor compliance to the DOT group was based on subjective assessment, it may have resulted in the accumulation of especially difficult-to-treat patients in the DOT group. Ultimately, we cannot exclude potential bias among the interdisciplinary team in judging patients’ adherence to DAA-therapy—however, it is not the intention of the authors to provide a standardized evaluation concept for therapy-adherence but to demonstrate the feasibility and effectiveness of DAA-treatment according to the concept of DOT in PWIDs with suspected poor compliance. The potential shortcomings of our evaluation system may lead to a broader inclusion into the DOT group than necessary, yet no disadvantages arise for patients who may have been assigned to the DOT concept despite excellent compliance. Similarly, the fact that IDU was assessed according to patients’ personal report may lead to an underestimation of ongoing IDU within the study population and may in fact contribute to an accumulation of especially difficult-to-treat PWIDs in the DOT group. However, this accumulation of PWIDs at especially high risk for non-adherence to DAA within the DOT group emphasizes the excellent results achieved by the applied DOT-concept. In fact, the results in the DOT group were identical to the results achieved in the comparison group, including patients without a history of IDU and PWIDs with presumed excellent compliance. Since it has been shown that treatment with DAA applied according to the concept of DOT leads to excellent SVR12 rates even in very-difficult-to-treat subgroups of PWIDs [15, 42, 43] and that DOT was well accepted by PWIDs while not posing any relevant changes in the patients’ daily routine as OAT-dispensation intervals were not changed, DOT was the preferred treatment setting in patients with questionable drug adherence. Furthermore, at the moment no standardized method to predict adherence to DAA treatment in difficult-to-treat PWIDs with ongoing IDU is available that is feasible in clinical settings [55]. From a scientific point of view, it would have been ideal to prove the superiority of the DOT-setting over the SS for PWIDs on OAT with a high risk for non-adherence to DAA by performing a randomized trial. However, we considered it unethical to conduct a randomized trial where PWIDs with a high risk of non-adherence to DAA-treatment could have been assigned to the SS.

One major strength of our study that underlines the positive impact of HCV-treatment according to the DOT-concept is the low rate of patients lost to follow-up: Overall, only 12/122 (9.8%) PWIDs treated in the DOT-setting were LTFU after the end of treatment while no patients were lost during treatment. This is not only relevant in terms of achieving high SVR-rates, but also beneficial from a socioeconomic point of view as less money is lost due to interrupted—and therefore ineffective—treatments. In the Viennese setting, raising physicians’ overall awareness towards HCV in PWIDs and providing an open-door-policy may be a promising approach to further increase SVR-rates by reducing LTFU-rates: In addition to scheduled HCV-surveillance, PWIDs may be tested for HCV-viremia when they are hospitalized or when they attend an outpatient clinic for other reasons than HCV-infection. It may be assumed that the implementation of point-of-care-tests for HCV-viremia at OAT-dispensing pharmacies would have reduced the LTFU-rate among our study population even further.

In conclusion, our results support the safety and effectiveness of HCV-treatment with SOF/VEL in PWIDs–including those with ongoing IDU–at high risk for non-adherence to therapy. By utilizing an integrative low-threshold drug treatment facility as an interface for screening and linkage to care for PWIDs on OAT, obstacles in HCV treatment were overcome and an effective strategy for directly observed antiviral therapy was established. No statistically relevant difference concerning the achievement of SVR could be detected between the DOT group and the SS group in this study. The DOT-concept proved to be highly effective as no major changes in the patients’ daily routine behavior were needed in order to facilitate treatment success and thus was well accepted by PWIDs. This microelimination strategy represents an effective measure in order to reach the WHO-goal of HCV-elimination by 2030 in Austria and may likely and easily be adopted by other countries.

## Supporting information

S1 Dataset (XLSX)

## Abbreviations

DAA: direct acting antivirals
HCV: hepatitis C virus
SVR: sustained virologic response
PWIDs: people who inject drugs
SOF/VEL: sofosbuvir/velpatasvir
OAT: opioid agonist therapy
IQR: interquartile range
GT: genotype
DOT: directly observed therapy
SS: standard setting
CI: confidence interval
FU: follow-up
WHO: World Health Organization
HIV: Human Immunodeficiency Virus
IDU: intravenous drug use
RNA: ribonucleic acid
TE: transient elastography
PCR: polymerase-chain-reaction
LTFU: lost to follow-up
